# A Study on Polymer Dispersants in CMP Ceria Slurry for the Polysilicon Film Removal Suppression

**DOI:** 10.3390/polym18192326

**Published:** 2026-09-24

**Authors:** Jaeyeong Cho, Seyoung Ahn, Sohee Hwang, Woonjung Kim

**Affiliations:** 1 Department of Nanomaterials and Semiconductor, Hannam University, Daejeon 34430, Republic of Korea; 2 Department of Chemistry, Hannam University, Daejeon 34430, Republic of Korea

**Keywords:** anionic polymer dispersant, CMP, STI, material removal rate (MRR), selectivity, Poly-Si removal suppression

## Abstract

CMP (Chemical Mechanical Polishing) is essential for achieving the desired surface quality and planarity required for subsequent layers and processing steps in semiconductor manufacturing. However, the aggregation of slurry particles caused by abrasive materials can lead to scratches, defects, and increased surface roughness, degrading the quality and durability of the finished surface during the CMP process. In this study, ceria slurry was prepared using an anionic polymer dispersant with an ammonium salt of an acrylate copolymer (AAPD) at different contents of 2.5, 3.0, 5.0, and 7.0 wt.% to minimize particle aggregation and to evaluate the polysilicon (Poly-Si) film removal suppression functionality commonly required in CMP processes. The CMP evaluation revealed that the HDP MRR (material removal rate) ranged from 580 to 660 Å/min at 60 s, while the Poly-Si MRR was significantly lower at 62 to 71 Å/min, confirming the existence of Poly-Si film removal suppression behavior. The substantially reduced Poly-Si MRR suggests that AAPD may contribute to polymer-mediated suppression of Poly-Si removal, although the specific inhibition mechanism remains to be clarified. This suggests that the AAPD enables the formulation of a one-component ceria slurry with effective Poly-Si film removal suppression capability for semiconductor CMP applications.

## 1. Introduction

Chemical mechanical polishing (CMP) is a universally recognized procedure for providing a global surface finish to different materials for a variety of applications in the semiconductor industry [1,2]. It is a surface smoothing process with the combination of controlled mechanical force, such as pressure or the relative motion of the polishing tool, and a chemical reaction for achieving the desired material removal [3,4,5]. As shown in Figure 1, CMP is a critical planarization process in semiconductor manufacturing, enabling the fabrication of multilayer device structures with nanometer-level precision [6].

Among the various CMP applications, STI (Shallow Trench Isolation) CMP is particularly important as it establishes electrical isolation between adjacent active regions in integrated circuits [7,8]. As shown in Figure 2, STI CMP Selectively planarizes the gap-fill oxide to produce a planarized surface. In STI CMP, a ceria (CeO_2_)-based slurry is widely employed owing to its high oxide MRR and superior selectivity compared to silica-based alternatives [9,10]. Ceria is recognized for its elevated oxide MRR owing to its strong chemical interaction with an oxide surface [11,12].

Various mechanisms explaining this interaction have been proposed by numerous researchers, with a consensus that the active sites for the reaction involve Ce^3+^ ions on the ceria surface [12,13]. The formation of an oxygen vacancy results in the reduction of cerium ions within the lattice, transitioning from Ce^4+^ to Ce^3+^. As shown in Figure 3, Ce^3+^ plays a pivotal role in initiating the reaction with the oxide surface, forming strong Ce–O–Si chemical bonds [12,13,14,15,16,17].

The evolution of semiconductor technology nodes has introduced increasingly demanding requirements for CMP performance. As device dimensions shrink, controlling selectivity among multiple materials, particularly silicon oxide (SiO_2_), silicon nitride (Si_3_N_4_), and Poly-Si has become essential [18,19]. As shown in Figure 4, in certain integration schemes, such as the construction of floating gates in NAND flash memory, the CMP process must effectively remove oxide while stopping on the underlying Poly-Si layer, requiring extremely high Oxide-to-Poly-Si selectivity [20].

Kim et al. demonstrated that a ceria slurry incorporating an anionic phosphate fluorosurfactant achieved an Oxide-to-Poly-Si selectivity of 295:1, enabled by the selective adsorption of the fluorosurfactant on the hydrophobic Poly-Si surface [20].

The mechanism by which additives suppress Poly-Si removal during CMP has been attributed to the formation of a passivation layer on the Poly-Si surface. Lee et al. reported that non-ionic surfactants such as Brij surfactants enhance oxide-to-polysilicon selectivity through a combined effect of adsorption and interfacial adhesion on the polysilicon surface; in particular, Brij52 with a low HLB value achieved a selectivity of 9.3, approximately 17 times higher than that of a conventional oxide slurry [21].

Park et al. proposed a multi-selectivity CMP approach using both anionic poly(acrylic acid) (PAA) and non-ionic poly(vinylpyrrolidone) (PVP) surfactants, where PVP selectively adsorbs on the Poly-Si surface to suppress its polishing rate [18].

The stability of the ceria slurry is another crucial concern for CMP applications. Agglomeration of ceria nanoparticles caused by high surface energy can lead to scratches, defects, and increased surface roughness, degrading the quality and durability of the polished surface [22,23]. Polymer dispersants play a vital role in maintaining colloidal stability by adsorbing onto particle surfaces and providing electrostatic and steric repulsion [24,25,26,27]. Hwang and Kim demonstrated that the incorporation of a defoaming polymer into the SMA-1000 dispersant polymer leads to superior thermal stability and increased uniformity, with a selectivity ratio of 80 for oxide and nitride films [28]. Furthermore, Hwang et al. reported that a zinc salt of ethylene acrylic acid (EAA) copolymer dispersant at 7.0 wt.% content (D7) yielded the smallest particle sizes and best long-term stability, owing to the effective adsorption of carboxyl groups (−COOH) onto the ceria particle surfaces [22].

Despite previous studies on surfactant- and polymer-mediated suppression of Poly-Si removal, these additives have mainly been investigated as dedicated inhibitors or passivating agents. In contrast, the present study investigates AAPD as the sole polymeric additive used primarily as a dispersant for ceria particles, while also evaluating its effect on Poly-Si removal suppression. This approach differs from our previous studies, which mainly focused on the colloidal or thermal stability of ceria slurries and oxide/nitride polishing behavior using other polymeric dispersants. Therefore, the novelty of this work lies in evaluating whether a ready-to-use AAPD formulation can simultaneously provide ceria dispersion and suppress Poly-Si removal without the addition of a separate Poly-Si inhibitor.

In this study, our objective is to develop a one-component ceria CMP slurry using AAPD as an anionic polymer dispersant at different contents of 2.5, 3.0, 5.0, and 7.0 wt.%. The goal is to overcome particle agglomeration, evaluate the Poly-Si film removal suppression functionality, and assess the long-term stability of the prepared slurry. Additionally, we aim to characterize the polishing rate efficiency of the slurry through the use of this anionic polymer dispersant.

## 2. Materials and Methods

### 2.1. Materials

Ceria (Solvay, Brussels, Belgium) particles were used as abrasive grains, and distilled water was used as the solvent for the CMP slurry preparation. As a reference, a commercially available ammonium methacrylate-based polymer dispersant (AMPD, Vanderbilt Minerals, LLC, Norwalk, CT, USA) was also used for comparison and is referred to as AMPD in this study. The chemical composition of the reference dispersant is ammonium polymethacrylate with a solid content of 25.0% and a density of 1.11 g/mL.

The dispersant employed in this study was an aqueous solution of an ammonium acrylate copolymer dispersant (AAPD, BYK-Chemie GmbH, Wesel, Germany), referred to as AAPD. The chemical composition of AAPD consists of 40–50% ammonium acrylate copolymer and 50–60% water, with a density of 1.16 g/mL.

### 2.2. Preparation of Ceria Slurry

Ceria slurries were prepared with ceria powder at 30 wt.% as the abrasive, and AAPD was added at 2.5, 3.0, 5.0, and 7.0 wt.% relative to the ceria mass on an active-polymer basis. The required amount of the AAPD product was calculated from its active-material content. The remainder of the slurry consisted of distilled water (Aquapuri Essence, Young In Chromass, Anyang, Republic of Korea). For the slurry preparation, ceria powder and the AAPD were combined in distilled water, and the mixture was subjected to basket milling (TEDI, Gunpo, Republic of Korea) to achieve the desired particle size reduction and uniform dispersion. The detailed formulation conditions of the slurries are summarized in Table 1. The milling process was conducted at 1500 rpm for 5 h using 0.8 mm zirconia beads, while maintaining the slurry temperature at 25 °C, with samples extracted at intervals (0 h, 1 h, 3 h, 5 h) to monitor the progression of particle size reduction and property changes.

### 2.3. Physicochemical Characterization of Dispersant and Ceria Slurry

FT-IR (Fourier-transform infrared) spectroscopy (Nicolet Summit X, Thermo Fisher Scientific, Waltham, MA, USA) was employed to identify the functional groups of the AMPD and AAPD. The FT-IR spectra of the liquid dispersant samples were collected over the range of 4000–400 cm^−1^ at a spectral resolution of 4 cm^−1^ with 32 scans per measurement. To evaluate the physicochemical properties of the prepared ceria slurry, a pH meter, and a conductivity meter (Orion Star A215, Thermo Fisher Scientific, Waltham, MA, USA) were employed. DLS (Dynamic light scattering, Litesizer 500, Anton Paar, Graz, Austria) was used for measuring particle size. Measurements were performed at 25 °C, with water selected as the dispersion medium in the Kalliope software (version 2.10.5, Anton Paar, Graz, Austria), with a viscosity of 0.8903 mPa·s. A zeta potential (Litesizer 500, Anton Paar, Graz, Austria) analysis based on electrophoretic light scattering (ELS) was conducted using an Omega cuvette (Litesizer 500, Anton Paar, Graz, Austria) at 25 °C to assess the electrostatic dispersion stability of the slurry particles. To observe the dispersion state and detect any agglomeration or precipitation, OM (optical microscopy, DN-10A, Samwon, Republic of Korea) analysis was employed at 400× magnification. For the characterization measurements, the ceria concentration was adjusted to 0.3 wt.% for DLS analysis, 3.0 wt.% for OM observation and zeta potential measurement, and 30.0 wt.% for pH and conductivity measurements.

### 2.4. Evaluation of the CMP

Polishing experiments of the prepared ceria slurries were conducted using a mini polisher (MetPrep 3^TM^ Grinder/Polisher, Allied High Tech Products, Inc., Cerritos, CA, USA) to evaluate the MRR. The final concentrations of ceria and polymer in the prepared ceria slurries during polishing were summarized in Table 2. HDP (high-density plasma) oxide, Nitride, and Poly-Si wafers were supplied by High-End Technology (Hwaseong, Republic of Korea). The initial film thicknesses of the HDP oxide, Nitride, and Poly-Si layers were 8000, 2000, and 2000 Å, respectively. No intentional surface treatment for native oxide removal was performed on the Poly-Si coupons prior to CMP. The native oxide state and surface hydrophobicity of the Poly-Si surface were not independently characterized in this study. Therefore, the discussion of the Poly-Si suppression behavior is based on the measured CMP performance rather than on an assumed hydrophobic surface state. The wafers were cut into 2.5 × 2.5 cm coupons and polished for 60 s using an IC1000 polyurethane polishing pad (DuPont, Wilmington, DE, USA) with a diamond-grit conditioner (3M Company, St. Paul, MN, USA). During CMP, the platen and carrier rotation speeds were set to 300 and 150 rpm, respectively, with an applied pressure of 4 psi and a slurry flow rate of 28.5 mL min^−1^. For each slurry condition, three independent polishing runs were performed using a new coupon for each run. Film thickness was measured before and after polishing using a thin-film thickness measurement system (ST4000, SJT, Daejeon, Republic of Korea). Prior to the measurements, the instrument calibration was verified using the dedicated calibration standard provided for the system. The measurement error of the thickness measurement instrument is 0.1 nm. For each coupon, film thickness was measured at nine positions within the central region of the 2.5 × 2.5 cm coupon. After polishing, the coupon was carefully repositioned in the same orientation and approximately the same location as in the pre-polishing measurement to evaluate comparable regions before and after CMP. Because positional fiducial markers were not used, the pre- and post-polishing measurement positions were regarded as approximately matched rather than exactly identical. The MRR for each polishing run was calculated from the difference between the mean pre- and post-polishing film thicknesses obtained from the nine measurement positions, divided by the polishing time. The reported MRR values are expressed as the mean ± standard deviation (SD) of three independent polishing runs (*n* = 3). The nine measurement positions within each coupon were used to evaluate the spatial variation in material removal. Because 2.5 × 2.5 cm coupon specimens rather than full wafers were used, this parameter is referred to as coupon-scale non-uniformity rather than within-wafer uniformity. Coupon-scale non-uniformity was calculated as Equation (1).

(1) $$Coupon-scale\;non-uniformity\left(\%\right)=\frac{Maximum-Minimum}{2\times Average}\times 100$$

The HDP/Nitride and HDP/Poly-Si selectivity were calculated as the ratios of the corresponding mean MRR values and reported to three significant figures. The Poly-Si film removal suppression functionality was assessed by comparing the Poly-Si MRR with the HDP and Nitride MRR. After polishing, AFM (atomic force microscope, nGauge, ICSPI Corp., Waterloo, ON, Canada) was employed to analyze the wafer surface topography and measure the surface roughness (Sa, arithmetical mean height), enabling evaluation of the polishing quality achieved with each slurry formulation. AFM measurements were performed with a scan size of 4.2 × 4.2 μm^2^ using a diamond tip with a tip radius of less than 20 nm. The central region of each coupon-scale wafer was scanned, and three-dimensional (3D) surface images were generated from the acquired AFM data using Gwyddion software (version 2.71). This study was conducted as a laboratory-scale screening test using coupon specimens.

### 2.5. Storage Stability of Ceria Slurry

The stability of the prepared ceria slurries was evaluated using 5 h milled slurries diluted from a ceria concentration of 30 wt.% to 4.07 wt.%. For the stability evaluation, the diluted slurry was divided into 12 vials and stored in ovens maintained at 25 °C and 60 °C for 6 weeks. Prior to each analysis or dilution, the samples were vortexed for 2 min to ensure redispersion, and DLS and zeta potential measurements were sampled from the middle of each vial. The DLS, zeta potential, pH, and conductivity were analyzed at weekly intervals, while OM was performed every 2 weeks to monitor changes in physicochemical properties over time. For the characterization measurements, the ceria concentration was adjusted to $4.07\times 10^{-2}$ wt.% for DLS analysis, $4.07\times 10^{-1}$ wt.% for OM observation and zeta potential measurement, and 4.07 wt.% for pH and conductivity measurements. The storage results characterize the colloidal stability of the diluted slurry intermediate and should not be interpreted as direct evidence of retained CMP performance at the final polishing concentration.

## 3. Results and Discussion

### 3.1. Characterization of the Dispersant

To compare the functional groups present in AMPD and AAPD, ATR-IR spectroscopy was performed. As shown in Figure 5, AMPD is an ammonium methacrylate-based polymer, whereas AAPD is commercially described as a solution of an ammonium acrylate copolymer. The detailed comonomer composition and molecular architecture of AAPD were not disclosed by the manufacturer. The FT-IR spectra of the two dispersants exhibited several common absorption bands, including an O-H/N-H stretching band near 3300 cm^−1^, a C-H stretching band near 2900 cm^−1^, an asymmetic carboxylate(COO^−^) stretching band near 1540–1550 cm^−1^, and a symmetic carboxylate(COO^−^) stretching band near 1410–1440 cm^−1^. These spectral similarities indicate that both dispersants contain common ammonium carboxylate-related functional groups. However, the similarity of the ATR-IR spectra does not establish that the two commercial polymers possess identical molecular architectures or differ only by a methyl substituent. Minor differences in peak intensity observed in the fingerprint region below 1500 cm^−1^ may arise from differences in polymer composition and molecular structure. These results indicate that AAPD contans ammonium carboxylate-related functional groups similar to those present in AMPD.

### 3.2. Evaluation of CMP Ceria Slurries with AAPD

The effect of milling time and dispersant concentration on the physicochemical properties of the ceria slurry with AAPD was investigated. The results are discussed in terms of DLS, zeta potential, pH, conductivity, and OM. The corresponding data are shown in Table 3.

As shown in Figure 6, the D50 particle size of ceria slurries is presented as a function of milling time for varying AAPD concentrations. The D50 particle size decreased dramatically from 1070 nm at the start to 251 nm after 5 h of milling for the AAPD 2.5 wt.%, representing a reduction of approximately 77%. It was confirmed that the basket milling process effectively breaks down large ceria agglomerates and achieves a uniform dispersion. After 5 h of milling, the final D50 values were 251 nm, 228 nm, 258 nm, and 224 nm for the 2.5, 3.0, 5.0, and 7.0 wt.% formulations, respectively. It was confirmed that all four dispersant concentrations achieved comparable particle size reduction, with the 7 wt.% formulation yielding the smallest D50 value. It is believed that the higher dispersant content facilitates more effective coverage of the ceria particle surfaces, enhancing electrostatic repulsion between particles and preventing re-agglomeration during and after milling. This observation is consistent with the findings of Hwang et al., who reported that increasing polymer dispersant content leads to decreased particle size due to more effective adsorption onto particle surfaces and increased electrostatic repulsion [22].

As shown in Figure 7, the zeta potential remained strongly negative throughout the milling process for all formulations. For the 2.5 wt.% formulation, the zeta potential ranged from −55.1 to −63.3 mV, indicating that the electrostatic stabilization was maintained consistently during the milling process. After 5 h of milling, the zeta potential values were −58.1 mV, −59.6 mV, and −58.5 mV for the 3.0, 5.0, and 7.0 wt.% formulations, respectively, demonstrating that all concentrations provide excellent electrostatic stabilization. It is believed that a zeta potential magnitude exceeding 30 mV is generally sufficient for a stable colloidal dispersion [29].

As shown in Figure 8, the pH increased during milling for all formulations. For the 2.5 wt.% formulation, the pH increased from 8.01 to 9.22. This increase may be associated with interactions between AAPD and the ceria particle surfaces during the milling process, rather than being attributed to a specific adsorption mechanism. Importantly, the pH at each milling stage decreased with increasing dispersant concentration: at the start, the pH values were 7.59, 7.24, and 6.85 for the 3.0, 5.0, and 7.0 wt.%, respectively. After 5 h of milling, the pH values increased to approximately 8.39–9.05, as the milling process promotes dispersant adsorption and equilibrates the ionic environment.

As shown in Figure 9, the conductivity showed a clear positive correlation with dispersant concentration, increasing from 3012 μS/cm at 2.5 wt.% to 9398 μS/cm at 7.0 wt.% after 5 h of milling. For the 2.5 wt.% formulation, the conductivity initially decreased from 4284 to 3068 μS/cm at 3 h before decreasing slightly further to 3012 μS/cm at 5 h. The decrease in conductivity may reflect changes in the ionic environment of the slurry during milling. This suggests that the conductivity of the ceria slurry was not excessively high after the milling process and therefore did not significantly affect the aggregation of the ceria particles.

As shown in Figure 10, OM images of the slurries at 400× magnification showed that at 0 h the particles were agglomerated and exhibited a non-uniform size distribution, whereas at 5 h the particles became uniform. This result was consistent with the particle sizes measured by DLS.

### 3.3. CMP Performance and Surface Characterization

The CMP performance of the ceria slurries prepared with different AAPD contents (2.5, 3.0, 5.0, and 7.0 wt.%) was evaluated using blanket wafers of HDP, Nitride, and Poly-Si films. The AMPD slurry prepared with a conventional ammonium polymethacrylate was also evaluated for comparison. The polishing was performed for 60 s under standard CMP conditions, and the MRR, coupon-scale non-uniformity, and selectivity were determined. The corresponding results are shown in Figure 11 and Table 4.

The HDP MRR of the AMPD slurry was 541 Å/min, while the AAPD slurries exhibited HDP MRR values ranging from 580 to 660 Å/min. Specifically, the AAPD 5.0 wt.% formulation showed the lowest HDP MRR at 580 Å/min, while the AAPD 3.0 wt.% formulation exhibited the highest at 660 Å/min. The HDP MRR values of all AAPD formulations were comparable to or higher than that of the AMPD, indicating that the AAPD does not adversely affect the oxide polishing capability of the ceria slurry. The non-monotonic variation in HDP MRR with increasing AAPD concentration may result from the combined effects of changes in ceria dispersion and polymer-mediated interfacial interactions during polishing. These competing effects may cause the abrasive–wafer interaction to vary with AAPD concentration rather than showing a simple concentration-dependent trend. Since direct adsorption measurements were not performed, this interpretation is proposed as a possible explanation rather than a confirmed mechanism.

The Nitride MRR showed more variation among the formulations. The AMPD slurry achieved a Nitride MRR of 164 Å/min, while the AAPD formulations exhibited Nitride MRR values of 165, 267, 189, and 198 Å/min for AAPD (2.5, 3.0, 5.0, and 7.0 wt.%), respectively. Notably, the AAPD 3.0 wt.% formulation showed the highest Nitride MRR at 267 Å/min, which is approximately 63% higher than that of the AMPD.

The most significant finding was observed in the Poly-Si MRR. While the AMPD slurry showed a Poly-Si MRR of 276 Å/min, all AAPD formulations exhibited dramatically lower Poly-Si MRR values of 62, 62, 71, and 68 Å/min for AAPD (2.5, 3.0, 5.0, and 7.0 wt.%), respectively. This represents a reduction of approximately 74–78% in Poly-Si MRR compared to the AMPD. This pronounced suppression of Poly-Si removal confirms the existence of Poly-Si suppression behavior induced by the AAPD dispersant. The substantial decrease in Poly-Si MRR suggests that AAPD may contribute to polymer-mediated inhibition of Poly-Si removal. A similar suppression of Poly-Si removal by an anionic phosphate fluorosurfactant was previously attributed to selective adsorption and passivation of the Poly-Si surface [20]. However, because direct adsorption measurements were not performed in this study, the specific inhibition mechanism of AAPD remains to be clarified.

The HDP coupon-scale non-uniformity values ranged from 6.5% to 13.4% across all formulations. The AAPD formulations showed a trend of decreasing HDP coupon-scale non-uniformity with increasing dispersant content, with the AAPD 7.0 wt.% formulation achieving the best HDP coupon-scale non-uniformity at 6.5%. It is believed that the higher dispersant content promotes more effective particle stabilization and uniform distribution across the wafer surface during polishing, thereby improving the HDP coupon-scale non-uniformity. For Nitride coupon-scale non-uniformity, the AMPD slurry showed 8.3%, while the AAPD formulations exhibited values ranging from 6.4% to 26%. The AAPD 3.0 wt.% formulation achieved the best Nitride coupon-scale non-uniformity at 6.4%, while the AAPD 5.0 wt.% and 7.0 wt.% formulations showed higher values at 26% and 19%, respectively. It is believed that the higher dispersant content may affect the slurry flow dynamics and pad–wafer interaction coupon-scale non-uniformity for the Nitride film, leading to increased coupon-scale non-uniformity. The Poly-Si coupon-scale non-uniformity showed considerable variation, with the AAPD formulations exhibiting values of 30%, 14%, 25%, and 22% for AAPD (2.5, 3.0, 5.0, 7.0 wt.%), respectively, compared to 9.7% for the AMPD. Because the average Poly-Si MRR of the AAPD formulations was substantially lower than that of the AMPD slurry, relatively small absolute differences in local removal can result in larger relative coupon-scale non-uniformity values. The observed variation may also be influenced by local differences in abrasive–surface contact, slurry distribution, and polymer-mediated inhibition during polishing.

The HDP/Nitride selectivity ranged from 2.47 to 3.81 for the AAPD formulations, compared to 3.30 for the reference. The AAPD 2.5 wt.% formulation showed the highest HDP/Nitride selectivity at 3.81, while the AAPD 3.0 wt.% formulation exhibited the lowest at 2.47, which is attributed primarily to its elevated Nitride MRR of 267 Å/min. Among the AAPD formulations, the AAPD 2.5 wt.% slurry exhibited a higher HDP/Nitride selectivity (3.81) than the AMPD (3.30), while the AAPD 7.0 wt.% formulation showed a slightly lower value (3.25). The AAPD 3.0 and 5.0 wt.% formulations yielded lower HDP/Nitride selectivity values of 2.47 and 3.07, respectively, while providing the additional benefit of Poly-Si suppression functionality.

As shown in Table 5, the HDP/Poly-Si selectivity showed the most dramatic difference between the AMPD and AAPD formulations. The AMPD slurry had an HDP/Poly-Si selectivity of only 1.96, indicating nearly comparable removal rates for HDP and Poly-Si which is unsuitable for applications requiring oxide removal with Poly-Si suppression. In contrast, the AAPD formulations achieved HDP/Poly-Si selectivity values of 10.2, 10.6, 8.17, and 9.46 for AAPD (2.5, 3.0, 5.0, 7.0 wt.%), respectively. This represents an approximately 4.2–5.4× improvement in HDP/Poly-Si selectivity compared to the AMPD. The AAPD 3.0 wt.% formulation showed the highest HDP/Poly-Si selectivity at 10.6, which is attributed to its combination of a relatively high HDP MRR (660 Å/min) and a low Poly-Si MRR (62 Å/min). Kim et al. demonstrated that a ceria slurry incorporating an anionic phosphate fluorosurfactant achieved an Oxide-to-Poly-Si selectivity of 295:1 [20]. While the selectivity values obtained in this study are lower than that reported by Kim et al., it should be noted that the present study employed a one-component slurry system without additional surfactant additives, which simplifies the formulation process and potentially improves slurry stability. These results demonstrate that the AAPD effectively provides Poly-Si suppression functionality in the ceria CMP slurry, enabling a one-component slurry system that achieves high Oxide-to-Poly-Si selectivity.

As shown in Figure 12, the surface quality after CMP was evaluated using atomic force microscopy (AFM). The surface roughness (Sa) of the polished wafers was measured for each film type across selected slurry formulations.

Prior to polishing, the Sa values were 0.068 nm for HDP, 0.070 nm for Nitride, and 0.071 nm for Poly-Si, confirming that all as-deposited films exhibited extremely smooth surfaces. Following polishing with the AMPD slurry, the Sa values were 1.667 nm for HDP, 1.355 nm for Nitride, and 1.434 nm for Poly-Si, indicating that smooth surfaces were maintained across all film types. For the HDP film, the AAPD formulations showed Sa values of 1.638, 1.597, 1.862, and 1.794 nm for AAPD (2.5, 3.0, 5.0, and 7.0 wt.%), respectively, comparable to those of the AMPD. For the Nitride film, the AAPD formulations showed Sa values of 1.454, 1.773, 1.871, and 1.749 nm for AAPD (2.5, 3.0, 5.0, and 7.0 wt.%), respectively, with the AAPD 2.5 wt.% formulation exhibiting a value similar to that of the AMPD, while the remaining formulations (3.0, 5.0, and 7.0 wt.%) showed comparable values within a similar range. For the Poly-Si film, the AAPD formulations showed slightly higher Sa values of 1.843, 1.796, 1.888, and 1.836 nm for AAPD (2.5, 3.0, 5.0, and 7.0 wt.%), respectively, compared with 1.434 nm for the AMPD. The slightly increased roughness may reflect differences in local abrasive–surface interactions and the substantially reduced Poly-Si removal under the AAPD conditions. However, the AFM roughness measurements alone do not provide direct evidence for polymer adsorption, passivation-layer coverage, or its spatial uniformity. Overall, the AFM results showed that the AAPD formulations maintained nanometer-scale surface roughness after polishing while exhibiting Poly-Si removal suppression.

### 3.4. Storage Stability Evaluation of Ceria Slurry

The six-week storage stability of the 4.07 wt.% ceria slurries was evaluated at 25 °C and 60 °C. DLS, zeta potential, pH, and conductivity were analyzed at weekly intervals, whereas OM observations were performed every two weeks. The stability results are discussed in terms of DLS, zeta potential, pH, conductivity, and OM.

At 25 °C, the D50 particle size of the AMPD slurry remained remarkably stable over the six-week period, ranging from 221.6 to 235.8 nm with no significant agglomeration trend, as shown in Figure 13. The AAPD 2.5 wt.% formulation also exhibited excellent stability, with D50 values ranging from 235.1 to 257.1 nm throughout the six weeks. It was confirmed that the lower content of the AAPD 2.5 wt.% formulation provided sufficient stabilization without significant particle growth, which is consistent with the finding that the dispersant achieved rapid equilibrium during the milling stage. In contrast, the AAPD (3.0, 5.0, and 7.0 wt.%) formulations showed an initial decrease in D50 during the first week of storage (to 189.6, 190.0, and 188.9 nm at 1 week, respectively), followed by a continued gradual decrease throughout the six-week period, reaching final D50 values of 158.3, 184.0, and 186.8 nm at 6 weeks.

Under the accelerated aging conditions at 60 °C, the D50 particle size results are summarized in Figure 13. The AMPD slurry maintained a stable particle size at 60 °C, with D50 values ranging from 219.8 to 232.9 nm, comparable to the 25 °C results. The AAPD2.5 wt.% formulation also showed excellent thermal stability, with D50 values ranging from 233.6 to 251.5 nm, again confirming that the lower-dispersant formulation is highly resistant to thermal agglomeration. AAPD (3.0, 5.0, and 7.0 wt.%) formulations exhibited a similar decreasing trend in D50 as observed at 25 °C, but the decrease was more pronounced at 60 °C. The AAPD 3.0 wt.% formulation showed the most significant decrease, reaching 140.5 nm in 6 weeks, while the AAPD 5.0, and 7.0 wt.% formulations decreased to 173.5 and 192.4 nm, respectively. A similar decrease in measured D50 was observed for the higher-AAPD formulations at 60 °C. No progressive increase in the measured hydrodynamic size was observed during the six-week period; however, DLS measurements of the sampled suspension alone cannot exclude preferential sedimentation or the loss of larger agglomerates.

The zeta potential values remained consistently negative throughout the six-week storage period at both 25 °C and 60 °C, as shown in Figure 14. At 25 °C, the AMPD slurry exhibited zeta potential values ranging from −53.6 to −60.1 mV, confirming stable electrostatic dispersion throughout the storage period. The AAPD 2.5 wt.% formulation showed zeta potential values between −52.0 and −54.0 mV during storage at 25 °C, remaining well within the stable range. The AAPD (3.0, 5.0, and 7.0 wt.%) formulations showed zeta potential values ranging from −50.5 to −61.0 mV, −54.3 to −60.6 mV, and −56.5 to −61.4 mV, respectively, at 25 °C. It is believed that a zeta potential magnitude exceeding 30 mV is generally sufficient for stable colloidal dispersion [29]. All formulations maintained zeta potential values well above this threshold throughout the six-week period at 25 °C, confirming that the electrostatic stabilization was not compromised during storage.

At 60 °C, the zeta potential values similarly remained strongly negative, ranging from −50.7 to −63.2 mV across all AAPD formulations, comparable to the 25 °C results. The reference slurry exhibited zeta potential values between −52.3 and −61.3 mV at 60 °C. The AAPD 2.5 wt.% formulation showed the lowest zeta potential magnitude at 5 weeks (−50.7 mV) among all samples at 60 °C; however, this value still exceeds the threshold for stable dispersion. The AAPD 3.0 wt.% formulation exhibited the highest zeta potential magnitude at 5 weeks (−63.2 mV). It was confirmed that the zeta potential of all formulations remained sufficiently high under accelerated aging conditions, indicating that the AAPD provides adequate electrostatic stabilization of ceria slurries even at elevated temperatures.

The changes in pH of the ceria slurries during the 6 weeks storage period at 25 °C and 60 °C are shown in Figure 15. At 25 °C, the reference slurry maintained a pH value between 8.52 and 9.15 over the six-week period; despite slight fluctuations, it remained alkaline. The AAPD 2.5 wt.% formulation exhibited the most stable pH at 25 °C, with values ranging from 8.95 to 9.31, showing minimal variation throughout the storage period. The AAPD 3.0 wt.% formulation showed pH values between 8.66 and 9.19, while the AAPD (5.0, and 7.0 wt.%) formulations exhibited lower pH ranges of 8.23–8.87 and 8.25–8.59, respectively. It was observed that higher dispersant concentrations corresponded to lower pH values.

At 60 °C, the pH showed a more pronounced decreasing trend compared to that at 25 °C, particularly for the higher dispersant concentration formulations. The AMPD slurry pH decreased gradually from 9.11 to 8.85 over 6 weeks. The AAPD 2.5 wt.% formulation maintained relatively stable pH values (8.85–9.34), showing the smallest overall decrease. However, the AAPD (5.0, and 7.0 wt.%) formulations showed significant pH decreases: the AAPD 5.0 wt.% slurry pH decreased from 8.40 to 7.85, and the AAPD 7.0 wt.% slurry decreased from 8.27 to 7.92 after 6 weeks. Despite these decreases, the pH remained within the alkaline range, and no adverse effects on slurry stability were observed, as evidenced by the consistently high zeta potential and stable particle size.

At 25 °C, the AMPD slurry exhibited conductivity values ranging from 385.7 to 494.7 μS/cm over the six-week period, which remained relatively low and stable. The AAPD 2.5 wt.% formulation showed conductivity values that stabilized in the range of 510.1–579.3 μS/cm through the six-week period. The AAPD 3.0 wt.% formulation exhibited a gradual increase in conductivity from 452.0 μS/cm at 1 week to 704.3 μS/cm at 6 weeks, while the AAPD (5.0, and 7.0 wt.%) formulations showed more significant increases from 820.1 to 1300.0 μS/cm and from 1205.3 to 1647.0 μS/cm, respectively. It was observed that the conductivity increase was proportional to the dispersant concentration, which is consistent with the higher concentration of free ammonium polyacrylate ions in the high-dispersant formulations.

At 60 °C, the conductivity values showed a similar increasing trend but with higher absolute values compared to those at 25 °C, as shown in Figure 16. The AMPD slurry conductivity increased from 389.6 to 556.4 μS/cm over 6 weeks. The AAPD 2.5 wt.% formulation maintained relatively stable conductivity values ranging from 509.6 to 565.0 μS/cm. The AAPD (3.0, 5.0, and 7.0 wt.%) formulations exhibited conductivity values of 484.2–800.2, 853.7–1529.3, and 1240.7–2083.3 μS/cm, respectively. It is important to note that despite the increasing conductivity trend, no significant particle aggregation was observed. These observations are consistent with maintained colloidal dispersion under the tested conditions, although they do not independently establish the underlying stabilization mechanism.

As shown in Figure 17, the OM images at 400× magnification were used as a qualitative screening method for large, optically visible aggregates and macroscopic sedimentation during the six-week storage test. No large visible aggregates or obvious precipitates were observed in the tested samples at either 25 or 60 °C. Therefore, the OM results are interpreted only as evidence for the absence of large, visible agglomerates or macroscopic sedimentation within the resolution of the optical microscope.

## 4. Conclusions

In this study, a ceria slurry was prepared using an ammonium acrylate copolymer at different contents of 2.5, 3.0, 5.0, and 7.0 wt.% to minimize the issue of particle aggregation commonly observed in CMP slurries, and the Poly-Si film removal suppression functionality was evaluated. All samples were characterized by particle size, zeta potential, pH, and conductivity to evaluate physicochemical properties, and the CMP performance and long-term stability were assessed.

It was confirmed that the particle size of the CMP slurry decreased with milling time, with D50 values of 251 nm, 228 nm, 258 nm, and 224 nm for the 2.5, 3.0, 5.0, and 7.0 wt.% formulations after 5 h of milling, respectively. Although the 7.0 wt.% formulation exhibited the lowest mean D50 value, no monotonic relationship between AAPD concentration and final particle size was observed. Thus, the present particle-size results do not identify 7.0 wt.% as a uniquely optimal formulation. Overall, the substantial particle-size reduction observed for all AAPD formulations indicates effective dispersion of the ceria particles during milling.

The CMP evaluation revealed the Poly-Si film removal suppression functionality of all prepared slurries. It was observed that the Poly-Si MRR were 62, 62, 71, and 68 Å/min for 2.5, 3.0, 5.0, and 7.0 wt.%, respectively, which are substantially lower than the HDP MRR values of 630, 660.0, 580, and 643 Å/min. The resulting HDP/Poly-Si selectivity values were 10.2, 10.6, 8.17, and 9.46, respectively. These results suggest that AAPD contributes to suppressing Poly-Si removal through a polymer-mediated mechanism. However, as direct adsorption and surface chemical analyses were not performed, the specific mechanism underlying the inhibition of Poly-Si removal remains to be elucidated.

The stability tests of the prepared slurries were evaluated at 25 °C and 60 °C for six weeks. It was observed that the particle sizes and zeta potential values remained stable at both temperatures. The pH showed a more pronounced decrease at 60 °C. The conductivity showed an increasing trend, but no significant aggregation or precipitation was observed in any formulation.

In conclusion, the AAPD enables the formulation of a one-component ceria slurry with effective Poly-Si film removal suppression capability. This suggests potential innovative advancements in slurry manufacturing and process efficiency for semiconductor CMP applications requiring high Oxide-to-Poly-Si selectivity. Further studies are recommended to investigate the optimization of dispersant concentration for specific device integration schemes.

## Figures and Tables

**Figure 1:**
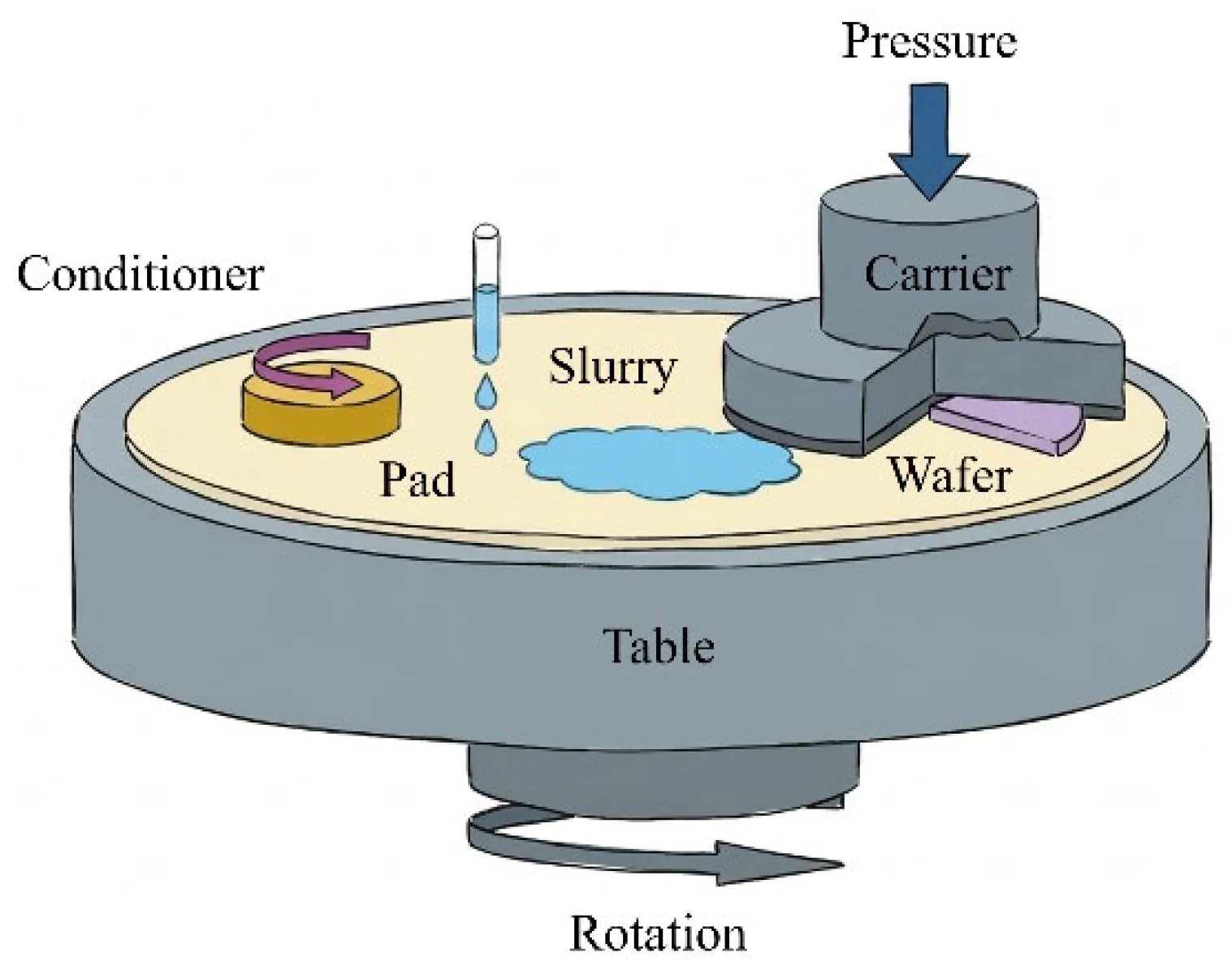
Schematic illustration of the chemical mechanical polishing (CMP) process.

**Figure 2:**
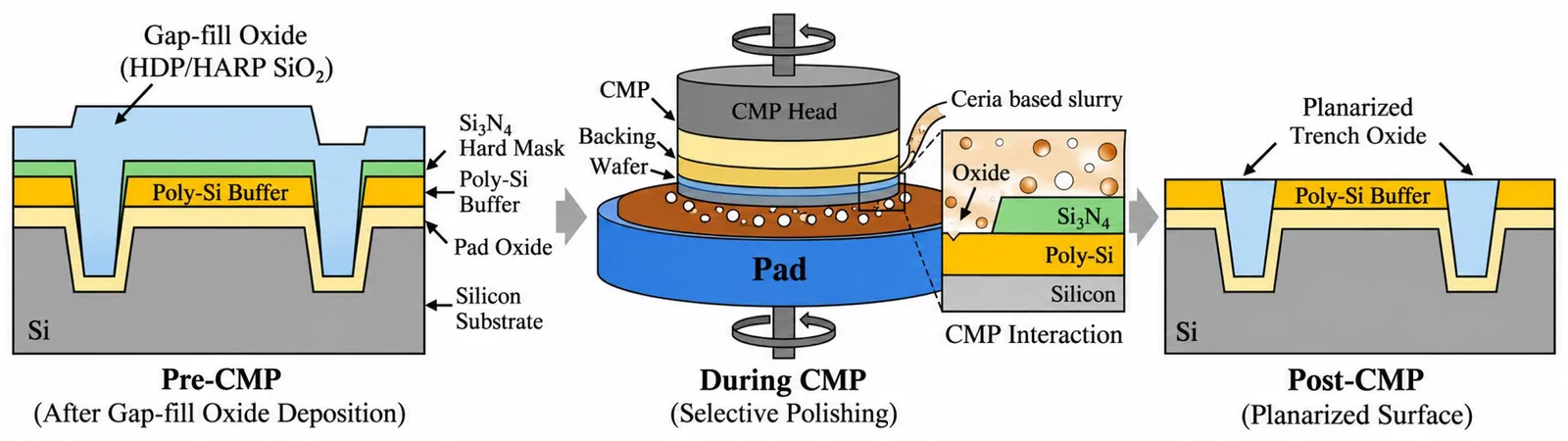
Schematic illustration of the oxide planarization during STI CMP using a ceria-based slurry.

**Figure 3:**
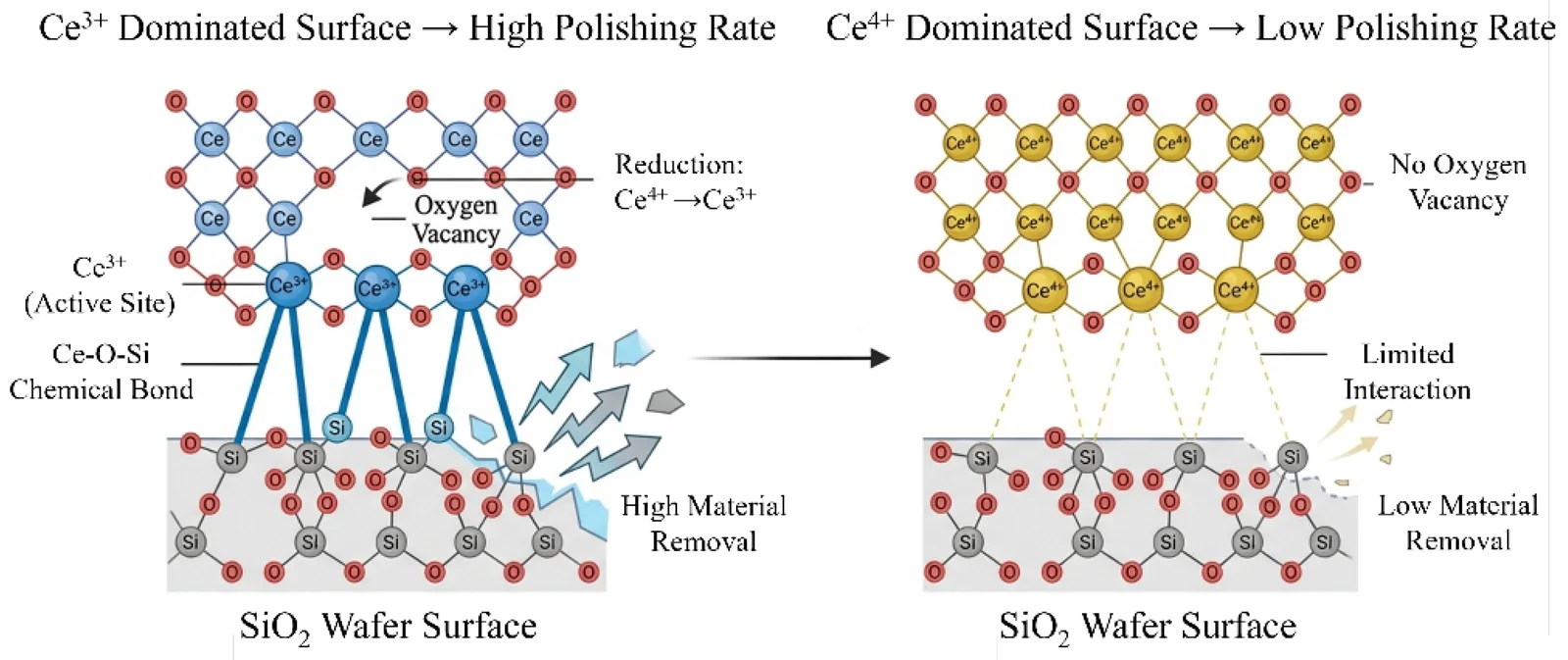
Schematic illustration of the relationship between the Ce^3+^/Ce^4+^ oxidation state and the wafer MRR during CMP.

**Figure 4:**
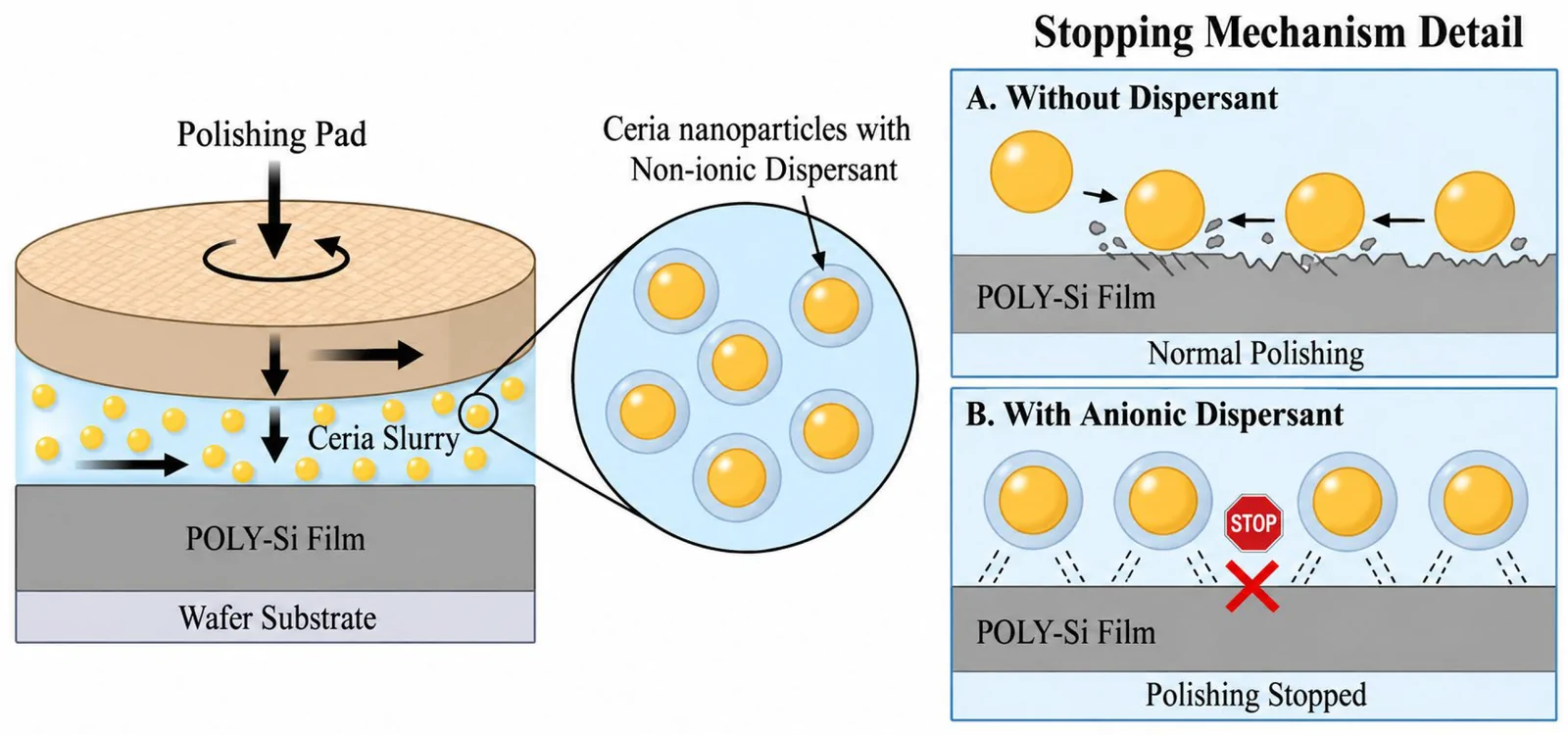
Schematic representation of the CMP process and the stopping mechanism of ceria slurry on Poly-Si film in the presence of an anionic dispersant.

**Figure 5:**
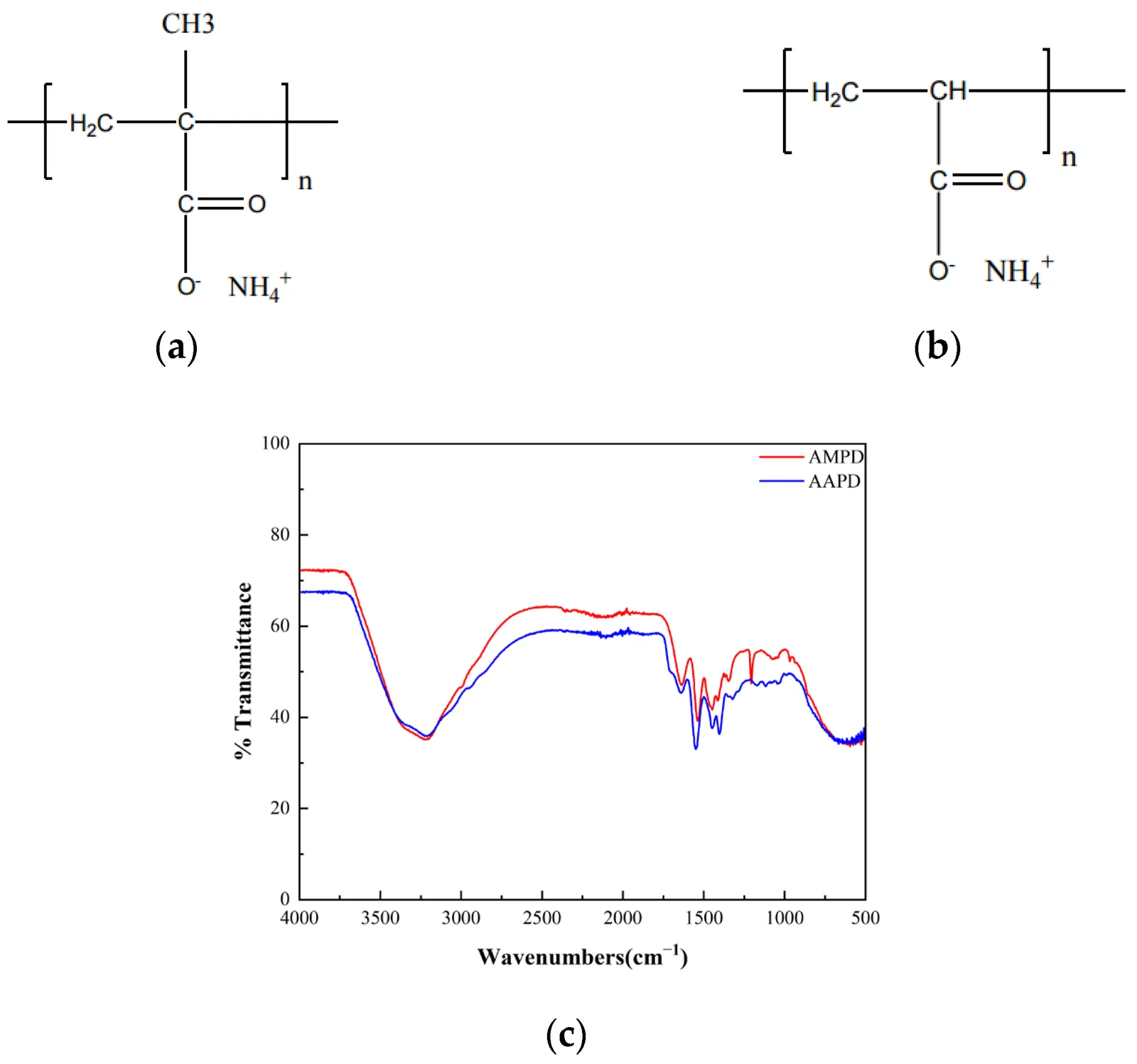
Chemical structures and FT-IR spectra of the AMPD and AAPD. (**a**) AMPD (**b**) AAPD (**c**) FT-IR spectra of the AMPD (red) and AAPD (blue).

**Figure 6:**
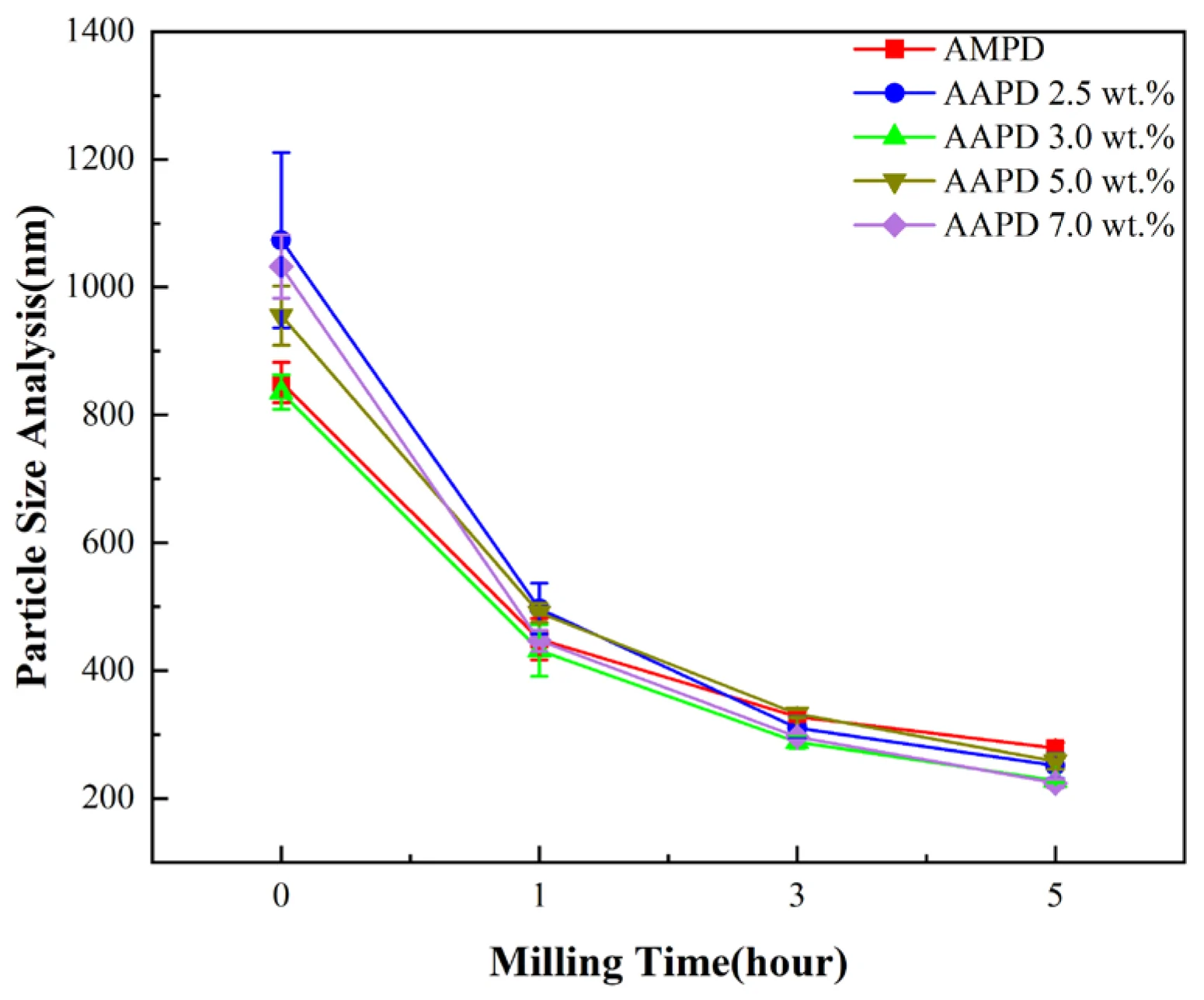
Particle size as a function of milling time for the AMPD slurry and AAPD containing slurries at various concentrations.

**Figure 7:**
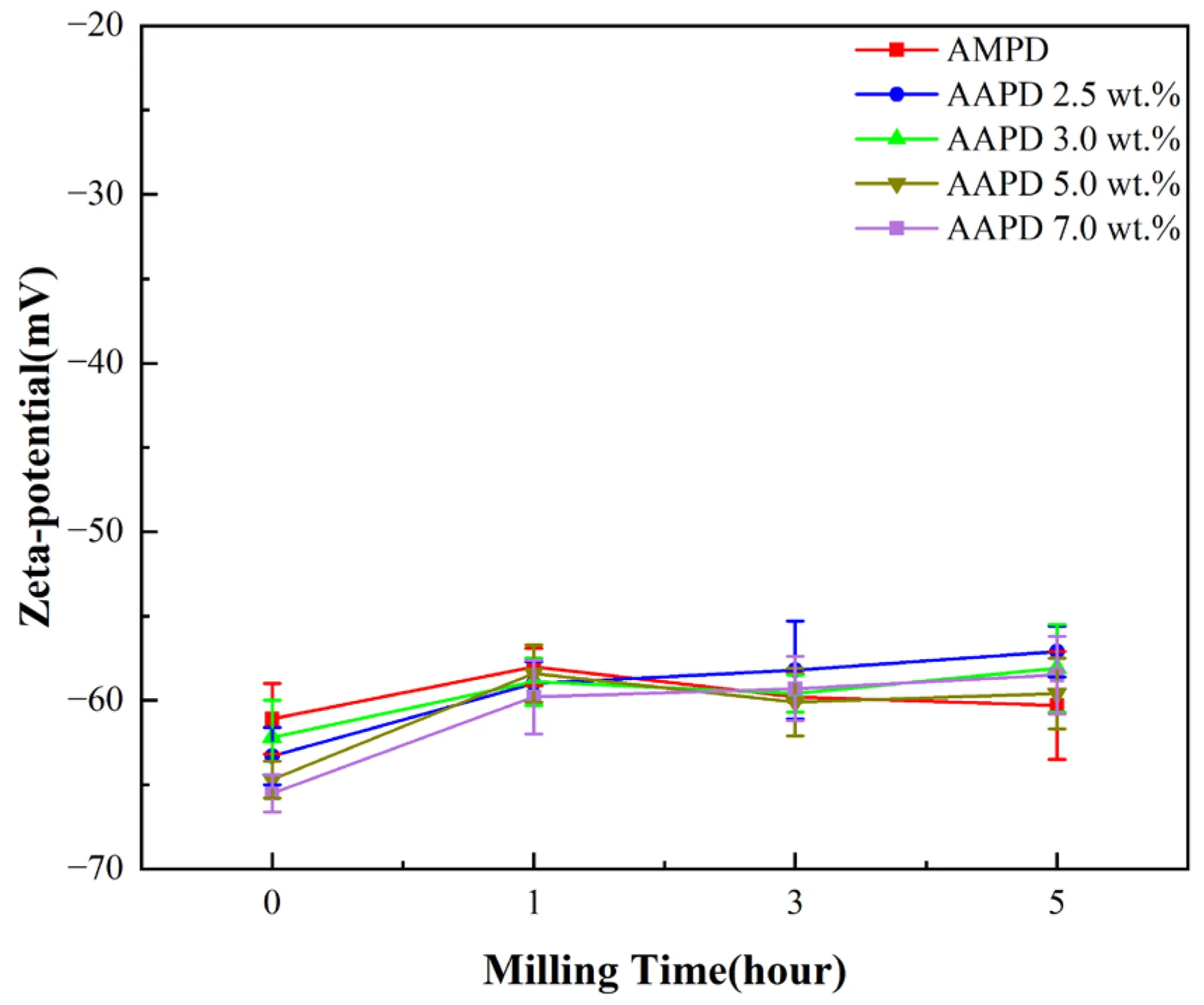
Zeta potential as a function of milling time for the AMPD slurry and AAPD containing slurries at various concentrations.

**Figure 8:**
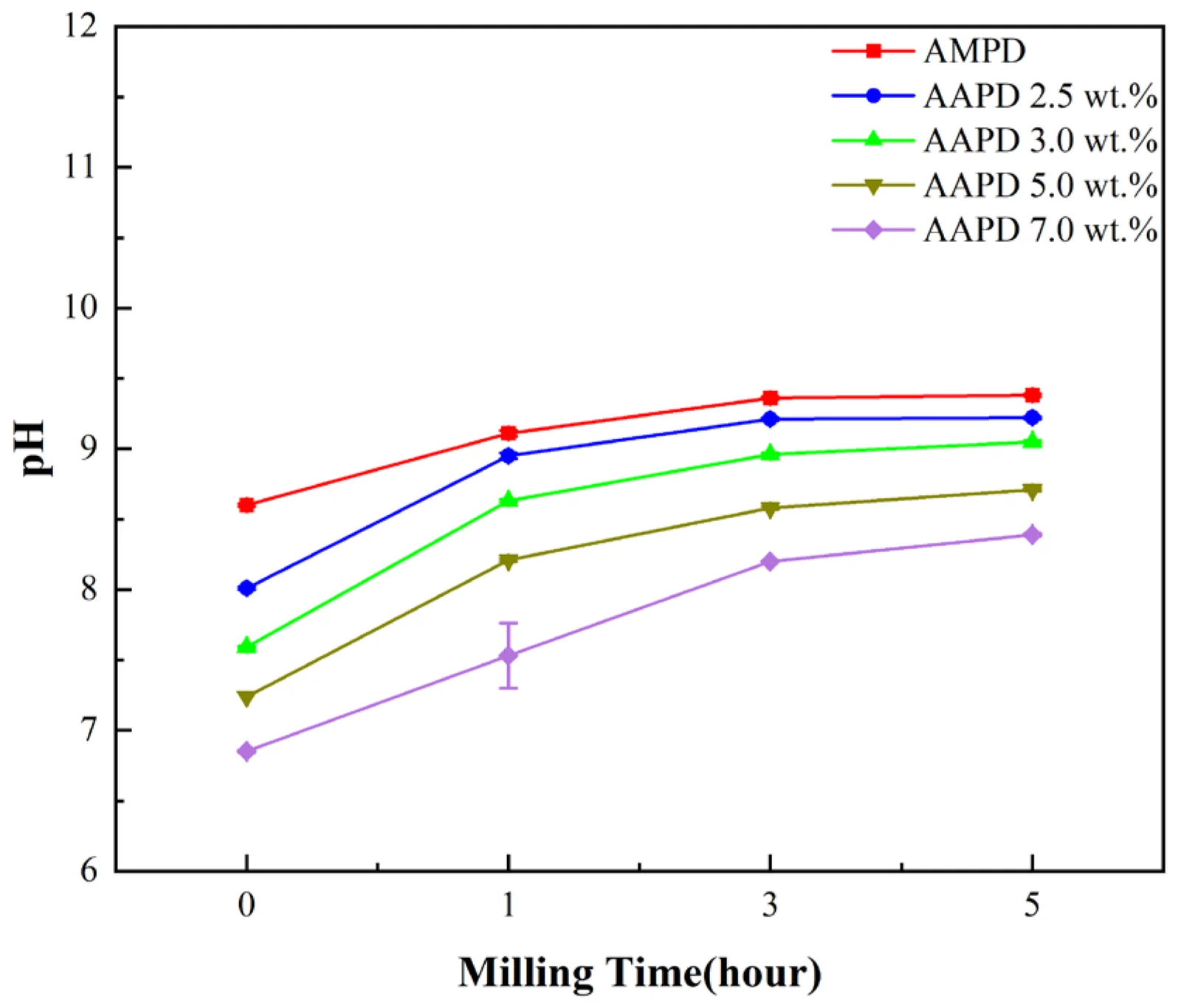
pH as a function of milling time for the AMPD slurry and AAPD containing slurries at various concentrations.

**Figure 9:**
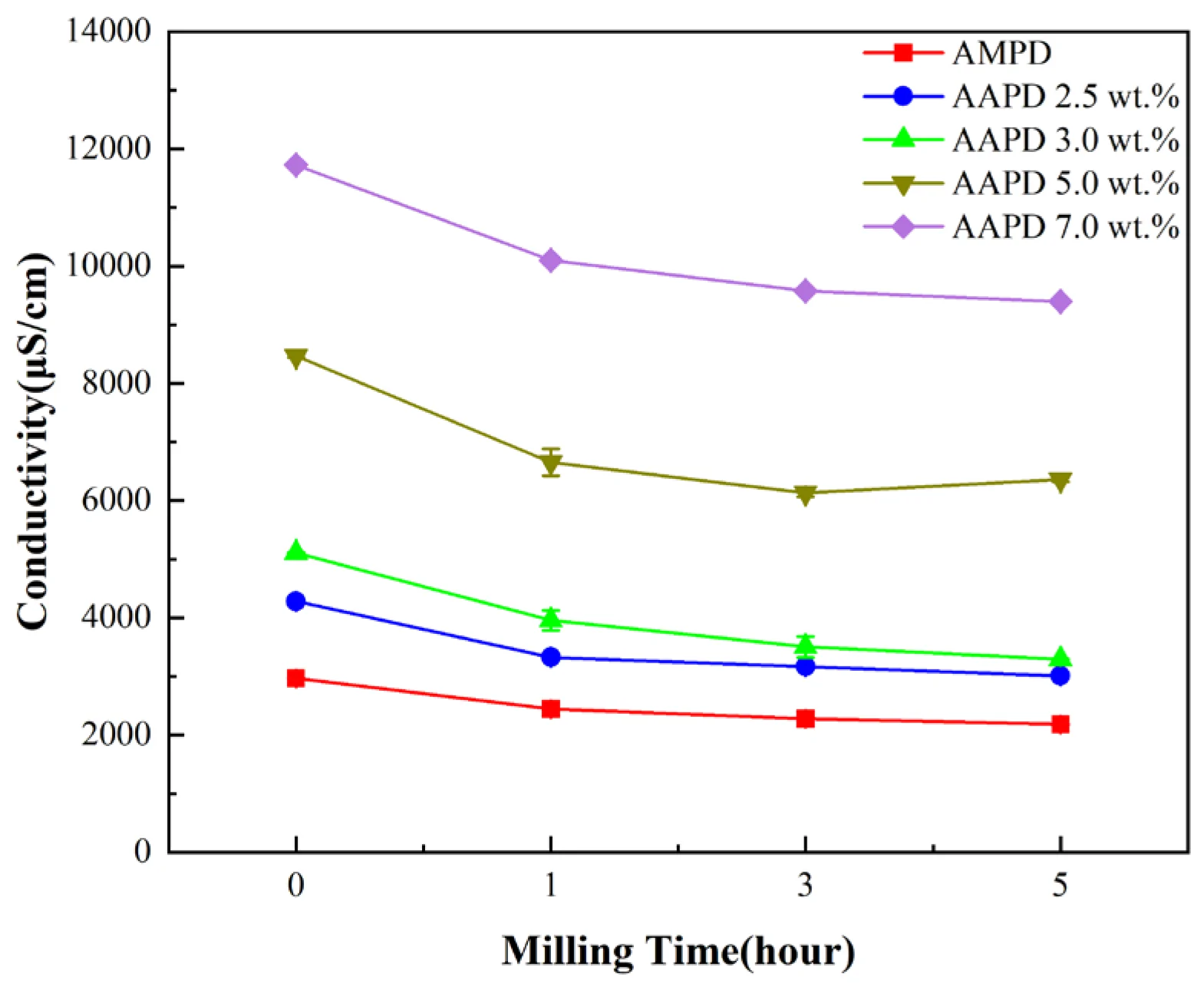
Conductivity as a function of milling time for the AMPD slurry and AAPD containing slurries at various concentrations.

**Figure 10:**
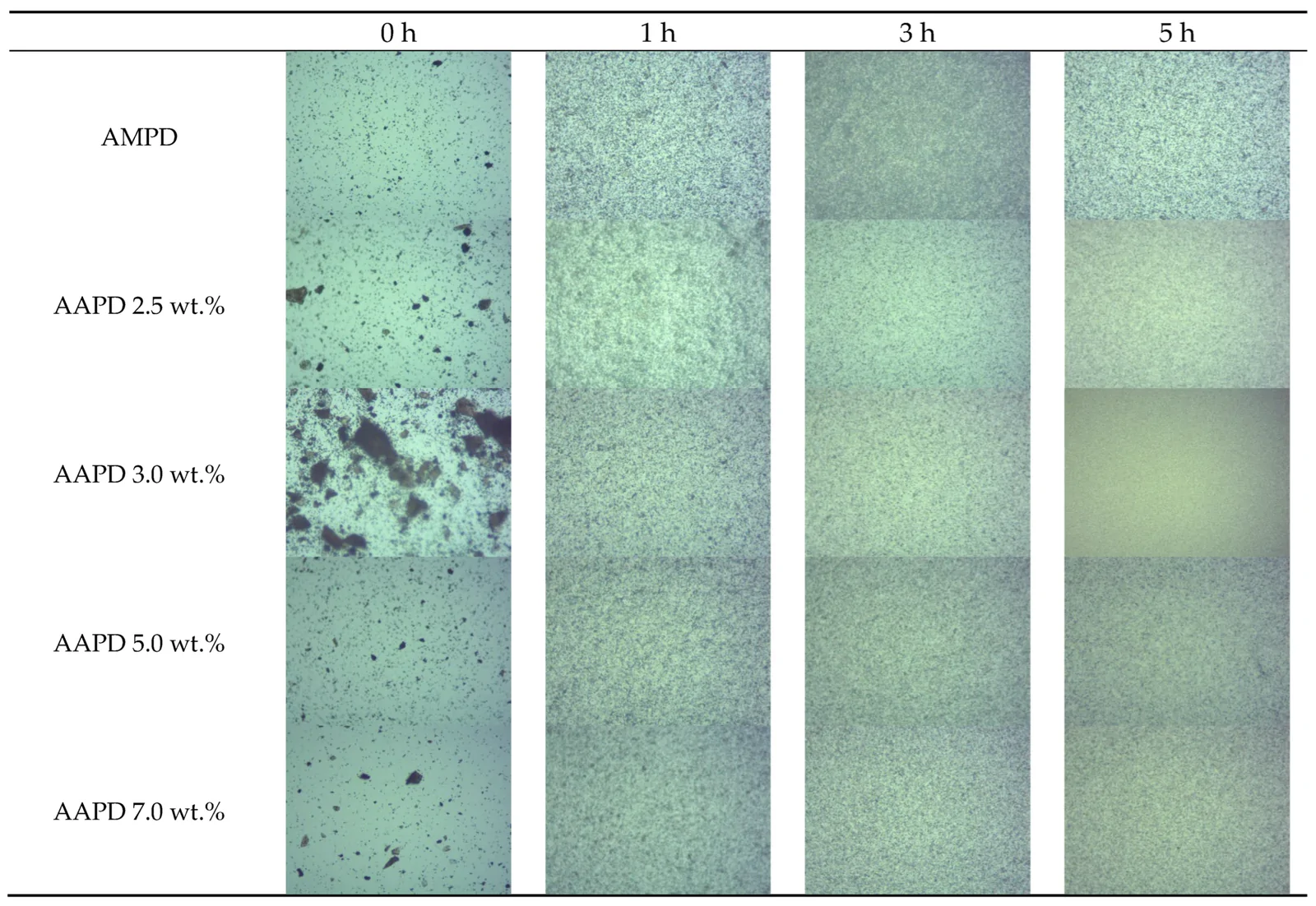
OM images (400×) as a function of milling time for the AMPD slurry and AAPD containing slurries at various concentrations.

**Figure 11:**
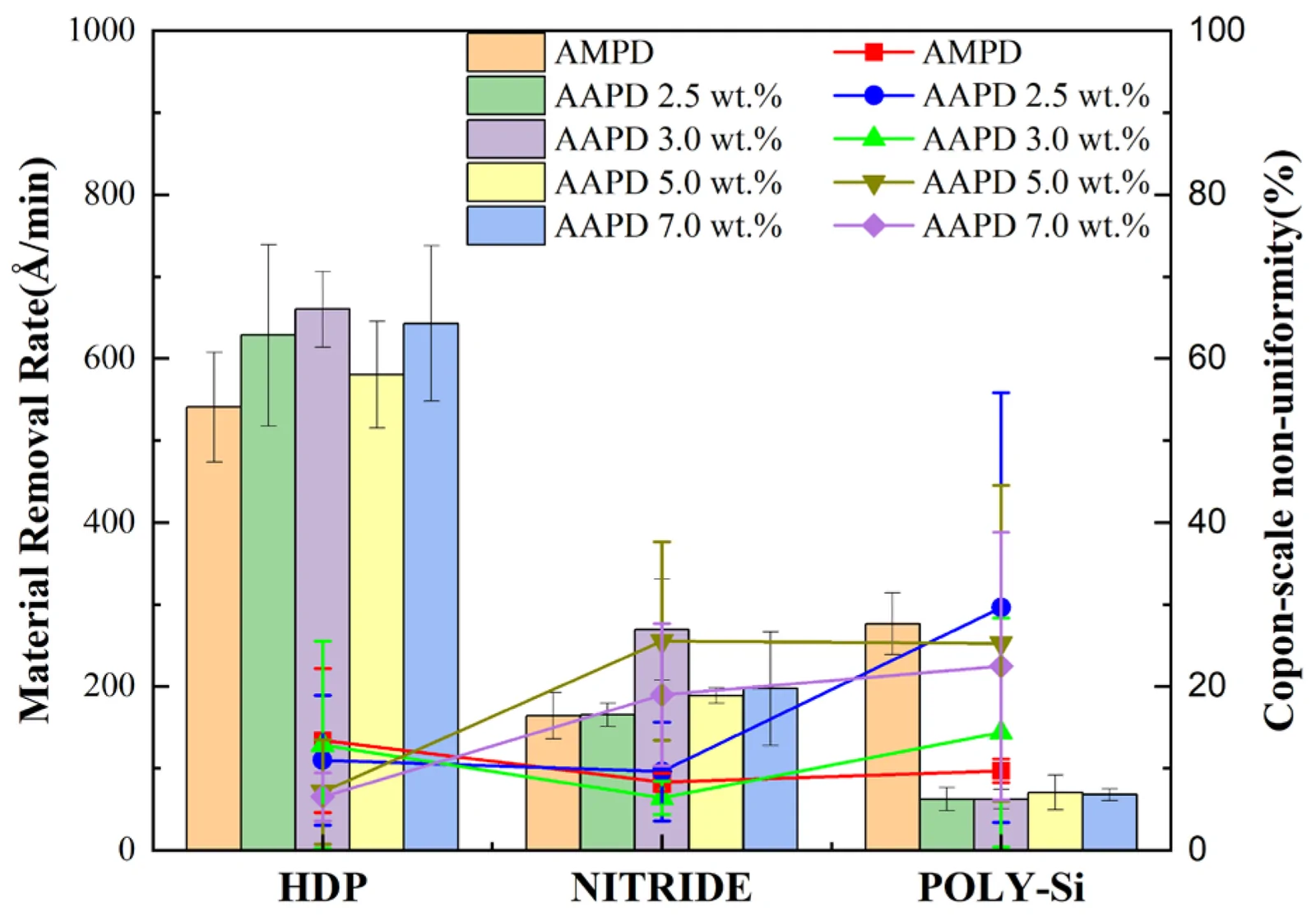
MRR (bars) and coupon-scale non-uniformity (lines) for HDP, Nitride, and Poly-Si films for the AMPD slurry and AAPD slurries at different concentrations.

**Figure 12:**
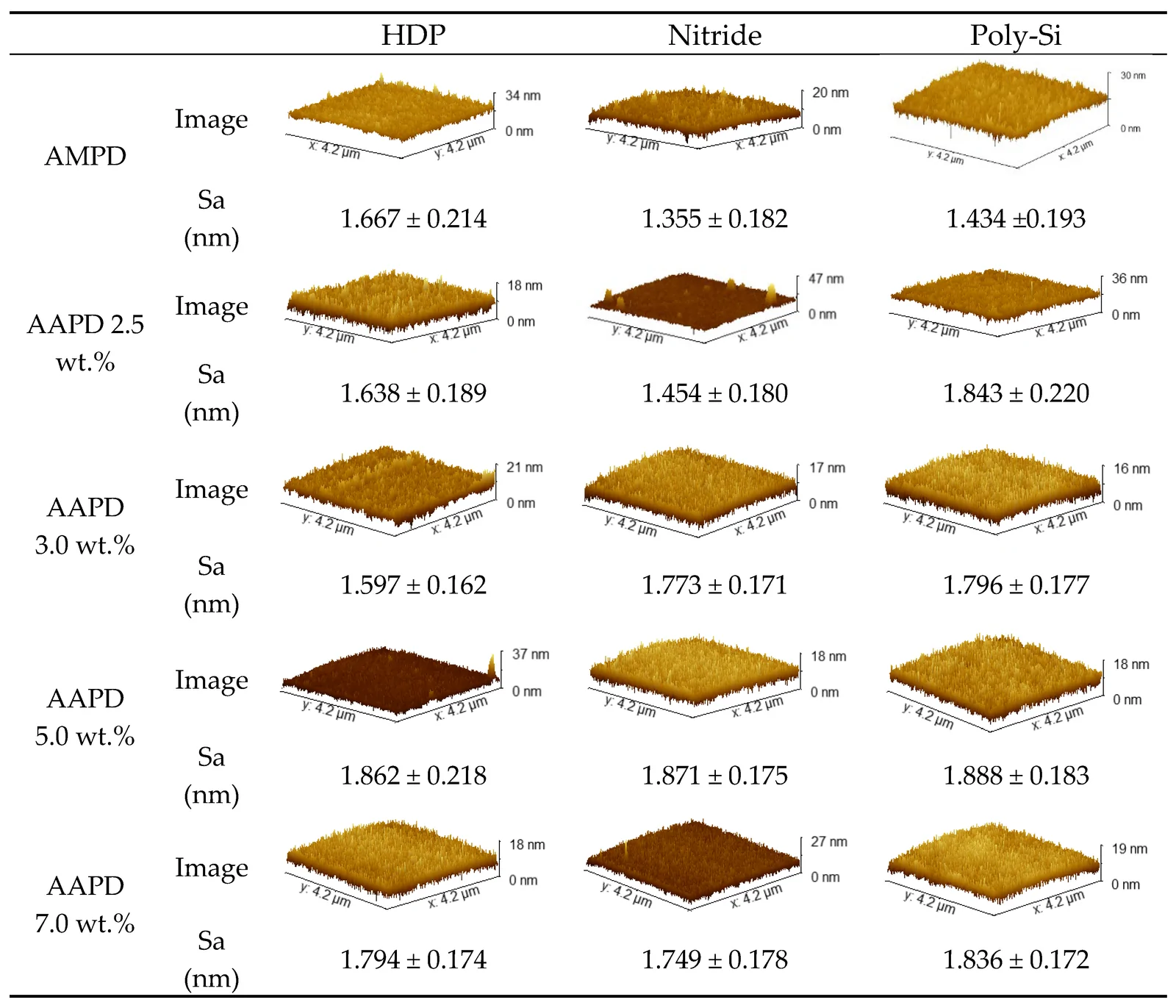
AFM images and surface roughness (Sa) of HDP, Nitride, and Poly-Si films polished with the AMPD slurry and AAPD slurries at different concentrations (Mean ± SD, *n* = 3).

**Figure 13:**
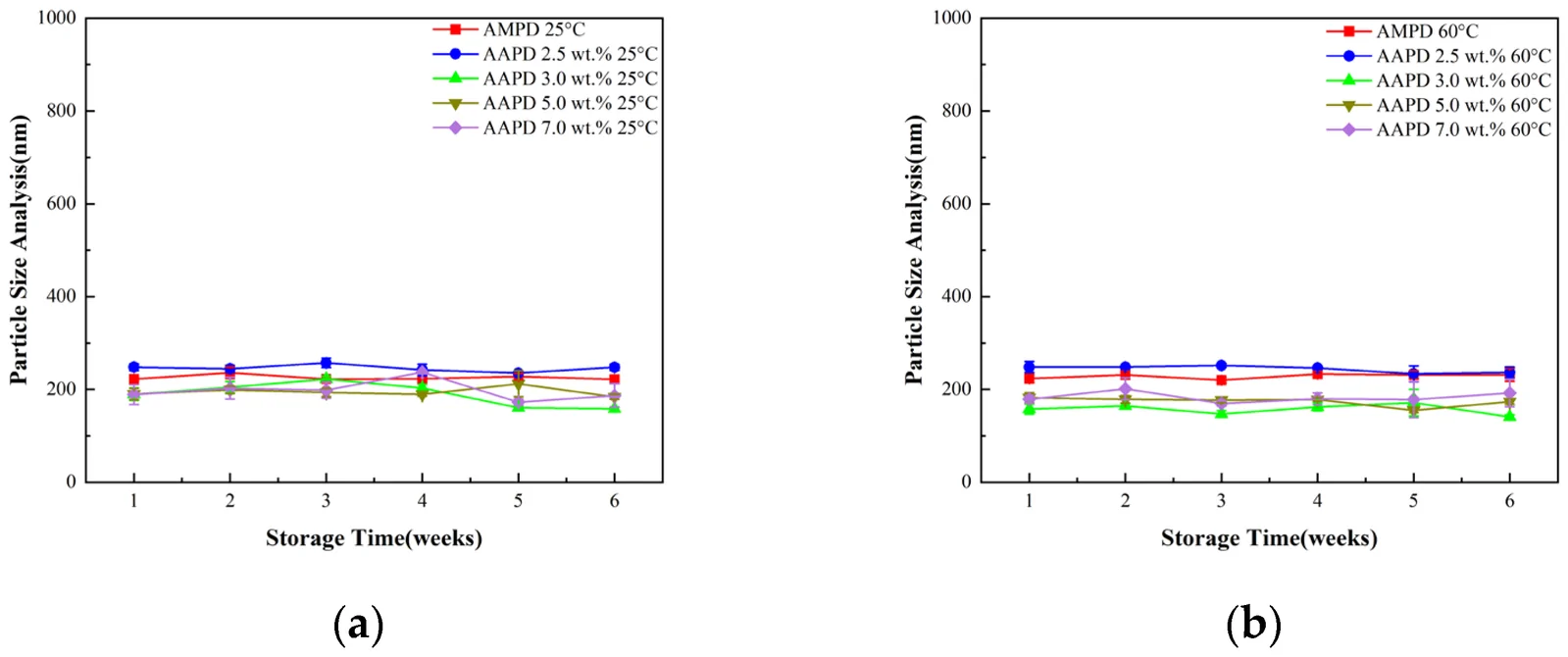
Particle size stability of the AMPD slurry and AAPD slurries at different concentrations as a function of storage time over 6 weeks at (**a**) 25 °C and (**b**) 60 °C.

**Figure 14:**
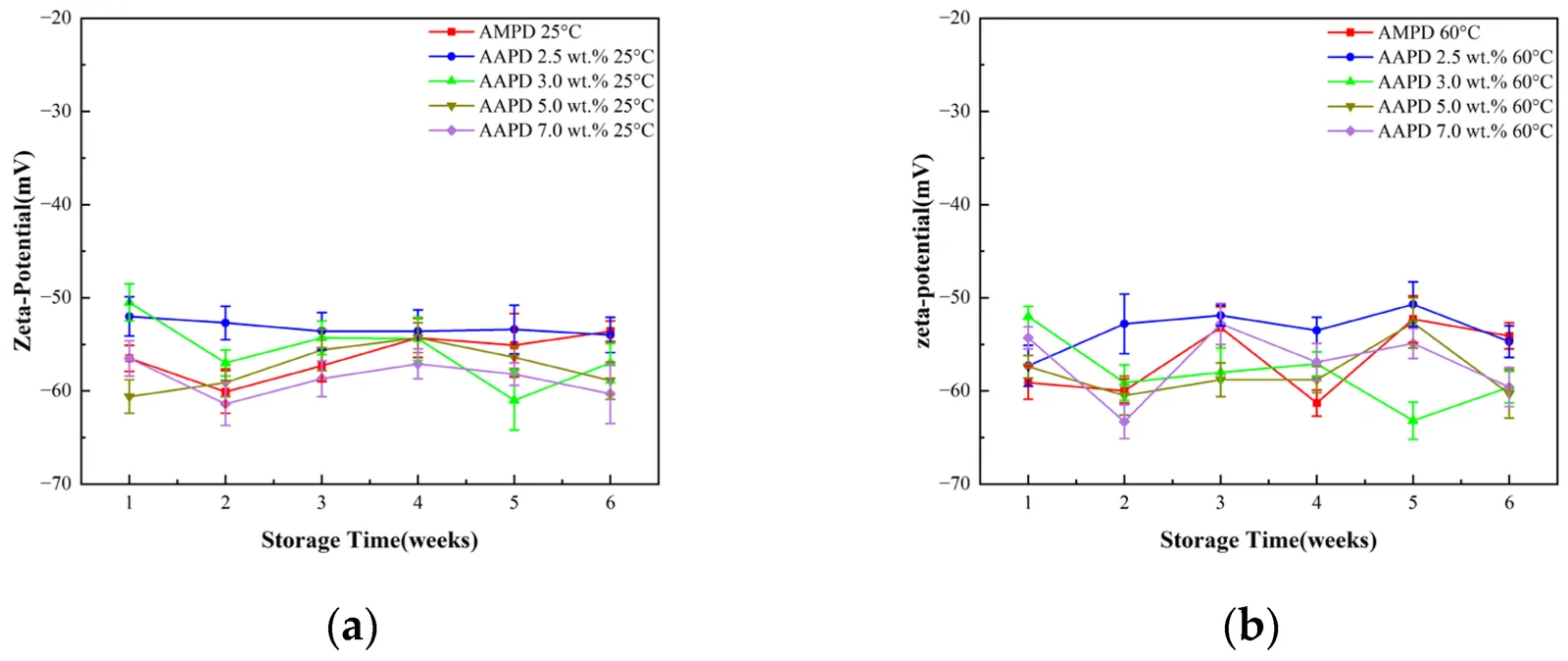
Zeta potential stability of the AMPD slurry and AAPD slurries at different concentrations as a function of storage time over 6 weeks at (**a**) 25 °C and (**b**) 60 °C.

**Figure 15:**
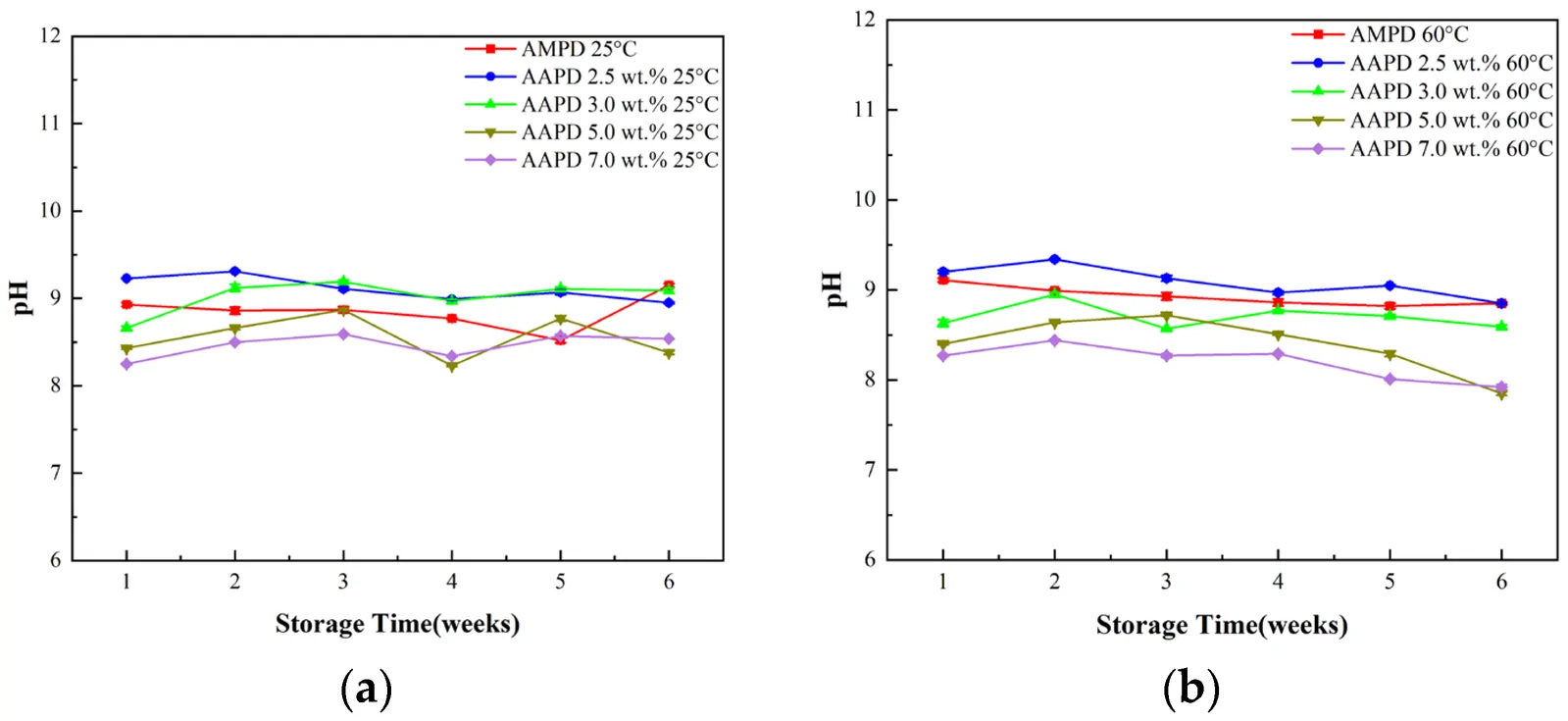
pH stability of the AMPD slurry and AAPD slurries at different concentrations as a function of storage time over 6 weeks at (**a**) 25 °C and (**b**) 60 °C.

**Figure 16:**
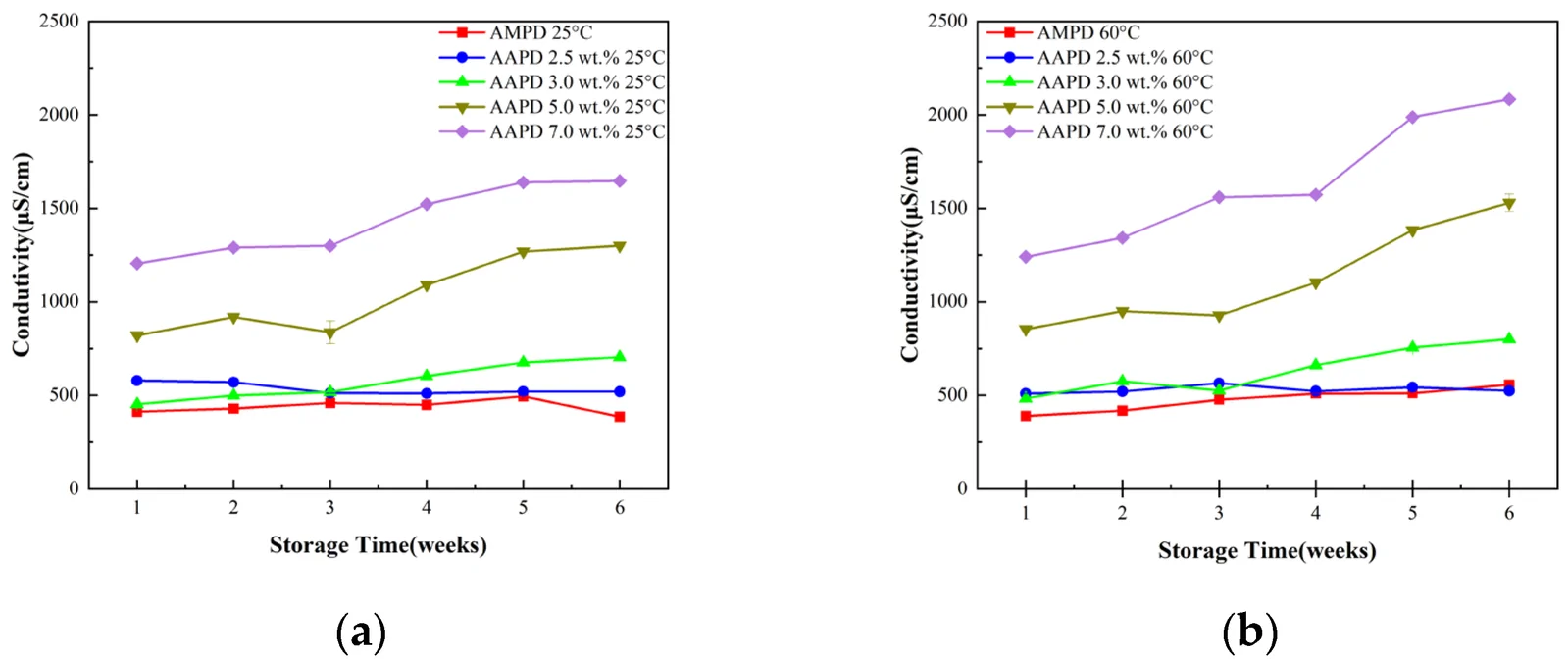
Conductivity stability of the AMPD slurry and AAPD slurries at different concentrations as a function of storage time over 6 weeks at (**a**) 25 °C and (**b**) 60 °C.

**Figure 17:**
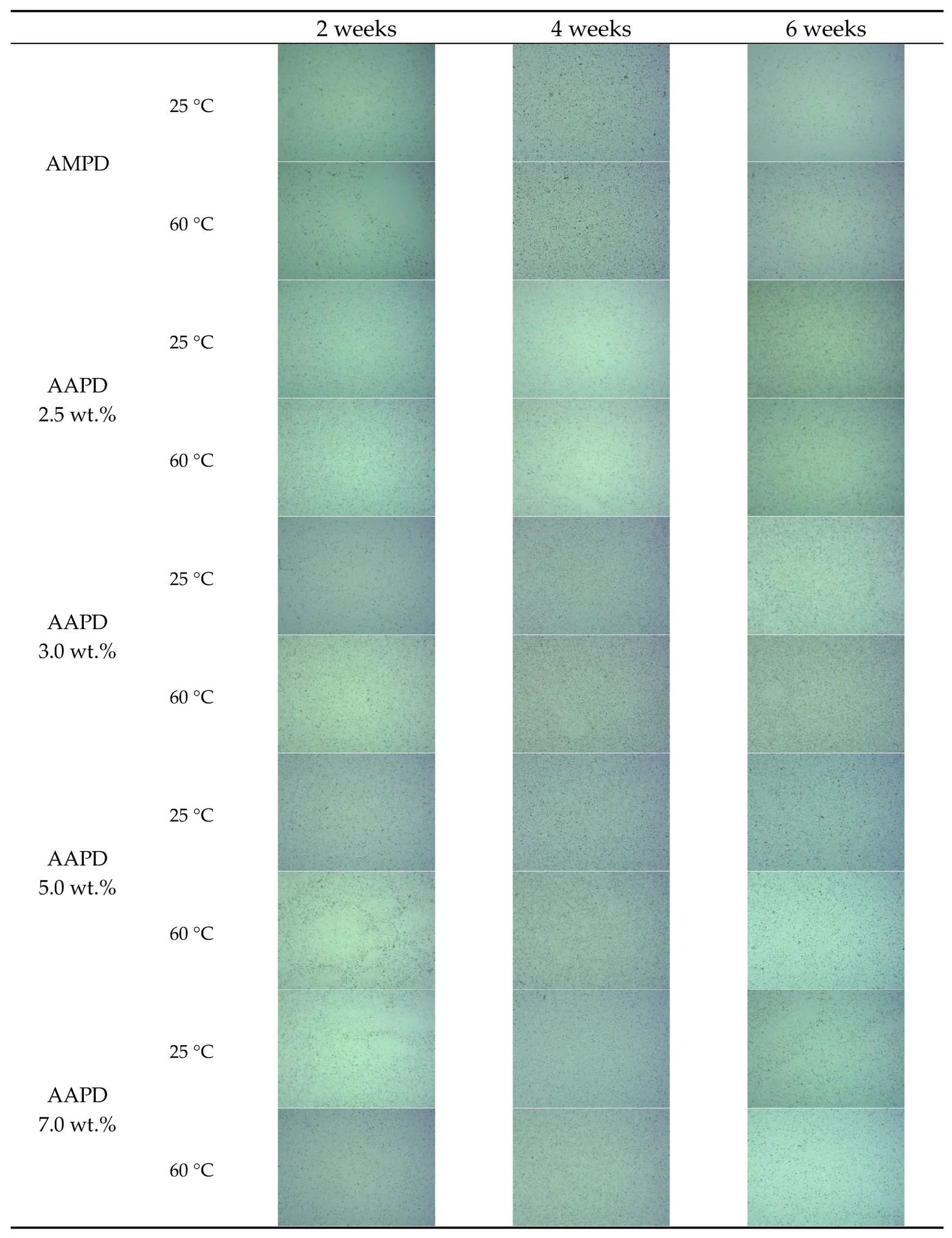
OM images (400×) of the AMPD slurry and AAPD slurries at different concentrations as a function of storage time over 6 weeks at 25 °C, and 60 °C.

**Table 1:** The formulation conditions of AMPD slurry and AAPD containing slurries at various concentrations (unit: g).

	Ceria	Dispersant		Distilled Water	Total
		Content	Active Polmer		
AMPD	180.0	18.0	4.5	402.0	600.0
AAPD 2.5 wt.%		10.7	4.5	409.3	
AAPD 3.0 wt.%		12.9	5.4	407.1	
AAPD 5.0 wt.%		21.4	9.0	398.6	
AAPD 7.0 wt.%		30.0	12.6	390.0	

**Table 2:** Concentrations of ceria and active polymer in the slurry formulations used for polishing (unit: wt.%).

	Ceria	Dispersant
AMPD	0.68	$1.70\times 10^{-2}$
AAPD 2.5 wt.%		$1.70\times 10^{-2}$
AAPD 3.0 wt.%		$2.05\times 10^{-2}$
AAPD 5.0 wt.%		$3.40\times 10^{-2}$
AAPD 7.0 wt.%		$4.75\times 10^{-2}$

**Table 3:** Physicochemical properties of the AMPD slurry and AAPD containing slurries at various concentrations (Mean ± SD, *n* = 3).

		Particle Size (nm)	Zeta Potential (mV)	pH	Conductivity (µS/cm)
AMPD	0 h	851 ± 31	−61.1 ± 2.1	8.60 ± 0.01	2970 ± 2
	5 h	279 ± 9	−60.3 ± 3.2	9.38 ± 0.01	2182 ± 3
AAPD 2.5 wt.%	0 h	1070 ± 140	−63.3 ± 1.7	8.01 ± 0.01	4284 ± 4
	5 h	251 ± 20	−57.1 ± 1.5	9.22 ± 0.01	3012 ± 8
AAPD 3.0 wt.%	0 h	836 ± 27	−62.2 ± 2.2	7.59 ± 0.01	5112 ± 4
	5 h	228 ± 3	−58.1 ± 2.6	9.05 ± 0.01	3291 ± 11
AAPD 5.0 wt.%	0 h	956 ± 46	−64.7 ± 1.1	7.24 ± 0.00	8468 ± 21
	5 h	258 ± 13	−59.6 ± 2.1	8.71 ± 0.01	6359 ± 37
AAPD 7.0 wt.%	0 h	1032 ± 49	−65.5 ± 1.1	6.85 ± 0.01	11,727 ± 6
	5 h	224 ± 7	−58.5 ± 2.3	8.39 ± 0.01	9398 ± 18

**Table 4:** MRR and coupon-scale non-uniformity of HDP, Nitride, and Poly-Si films for the AMPD slurry and AAPD slurries at different concentrations (Mean ± SD, *n* = 3).

		HDP	Nitride	Poly-Si
AMPD	MRR (Å/min)	541 ± 67	164 ± 28	276 ± 38
	coupon-scale non-uniformity (%)	13.4 ± 8.8	8.3 ± 1.1	9.7 ± 1.4
AAPD 2.5 wt.%	MRR (Å/min)	630 ± 110	165 ± 14	62 ± 14
	coupon-scale non-uniformity (%)	11.0 ± 7.9	9.6 ± 6.0	30.0 ± 26.0
AAPD 3.0 wt.%	MRR (Å/min)	660 ± 46	267 ± 62	62 ± 12
	coupon-scale non-uniformity (%)	13.0 ± 13.0	6.4 ± 2.0	14.0 ± 14.0
AAPD 5.0 wt.%	MRR (Å/min)	580 ± 65	189 ± 9	71 ± 21
	coupon-scale non-uniformity (%)	7.1 ± 6.4	26.0 ± 12.0	25.0 ± 19.0
AAPD 7.0 wt.%	MRR (Å/min)	643 ± 95	198 ± 69	68 ± 7
	coupon-scale non-uniformity (%)	6.5 ± 2.9	19.0 ± 8.7	22.0 ± 16.0

**Table 5:** Selectivity of HDP, Nitride, and Poly-Si films for the AMPD slurry and AAPD slurries at different concentrations.

	AMPD	AAPD 2.5 wt.%	AAPD 3.0 wt.%	AAPD 5.0 wt.%	AAPD 7.0 wt.%
HDP/Nitride	3.30	3.81	2.47	3.07	3.25
HDP/Poly-Si	1.96	10.2	10.6	8.17	9.46

## Data Availability

Data are available from the corresponding author upon reasonable request.
